# Metabolic analysis of mouse bone-marrow-derived dendritic cells using an extracellular flux analyzer

**DOI:** 10.1016/j.xpro.2021.100401

**Published:** 2021-03-26

**Authors:** Kazuhito Gotoh, Yurie Takata, Yuya Nakashima, Soichi Mizuguchi, Keishi Komori, Dongchon Kang

**Affiliations:** 1 Department of Clinical Chemistry and Laboratory Medicine, Graduate School of Medical Sciences, Kyushu University, 3-1-1, Maidashi, Higashi-ku, Fukuoka 812-8582, Japan

**Keywords:** Cell biology, Cell culture, Cell isolation, Cell-based assays, Immunology, Metabolism

## Abstract

Dendritic cell (DC) maturation induced by Toll-like receptor (TLR) agonists requires the activation of downstream metabolic changes. Here, we provide a detailed protocol to measure glycolysis, mitochondrial respiration, and fatty acid oxidation in mouse bone-marrow-derived DCs with the Seahorse XF24 extracellular flux (XF) analyzer. XF analysis with the Seahorse bioanalyzer has become a standard method to measure bioenergetic functions in cells, and this protocol can be adapted to other immune cells.

For complete information on using this protocol, please refer to Gotoh et al. (2018).

## Highlights

• Measuring energy metabolism in dendritic cells with an extracellular flux analyzer
• Analyzing TLR-induced glycolytic changes in dendritic cells
• Analyzing mitochondrial stress test and fatty acid oxidation in dendritic cells

## Graphical Abstract

**Figure:**
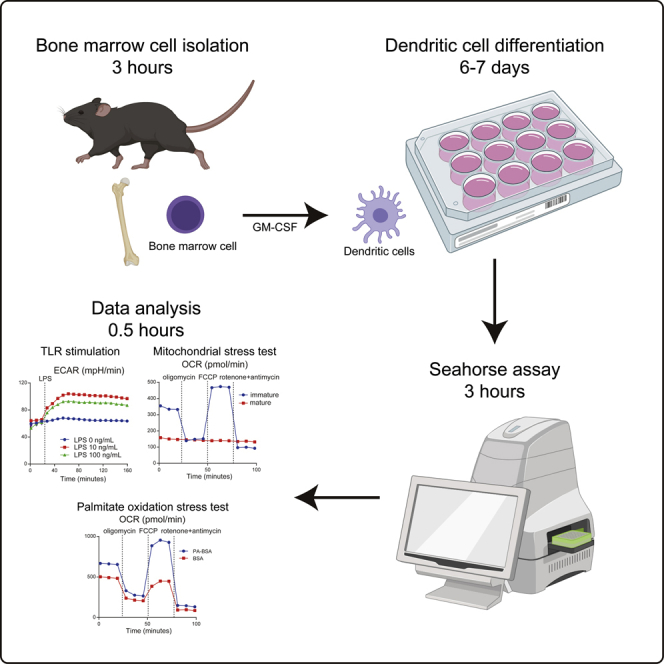

## Before you begin

Immunometabolism is an emerging field of investigation at the interface between the historically distinct disciplines of immunology and metabolism (Mathis and Shoelson, 2011). Recent studies on immunometabolism in myeloid dendritic cells (mDCs) provide new insights on the mechanism of the critical controllers of innate and adaptive immunity (Pearce and Everts, 2014) (O'Neill and Pearce, 2015). In particular, extracellular flux (XF) analysis has become a standard method to measure bioenergetic functions in DCs (Everts et al., 2012) (Everts et al., 2014) (Pantel et al., 2014) (Pelgrom et al., 2016).

This protocol comprises several methods to quantify the energy utilization of DCs in real-time using a Seahorse extracellular flux analyzer. The basic protocol describes a standard test with an XFe24 analyzer. If you use an XFe96 analyzer, you should adjust the number of DCs and the volume of buffer used according to the XFe96 analyzer. Please check the latest information on the Agilent website (https://www.agilent.com/en/product/cell-analysis/real-time-cell-metabolic-analysis) before your experiments.

In this protocol, we mainly described the analytical methods using mouse bone marrow-derived DCs (BMDCs). If you are using other cell types, please refer to Table 1. Although we show the number of cells that we could analyze so far in Table 1, we recommend that you consider the appropriate number of cells before starting the experiments.

**CRITICAL:** To obtain better results, it is important to seed more cells by monolayers

**Table 1:** Proposed seeding densities for different cell types in an XF 24-well plate

Cells	Cells/well	Medium	Reference
Mouse bone marrow derived dendritic cells	200,000	RPMI	(Gotoh et al., 2018)
Mouse splenic dendritic cells	200,000	RPMI	(Gotoh et al., 2018)
Human monocyte derived dendritic cells	200,000	RPMI	n/a
Mouse embryonic fibroblasts (MEF)	20,000	DMEM	(Monji et al., 2016)
Mouse primary neurons	40,000	DMEM	(Yagi et al., 2017)
Mouse primary oligodendrocytes	40,000	DMEM	(Yagi et al., 2017)
Mouse natural killer cells	200,000	RPMI	n/a
Mouse CD8 lymphocyte	800,000	RPMI	n/a
Mouse peritoneal macrophages	80,000	RPMI or DMEM	n/a
Mouse bone marrow derived macrophages	80,000	RPMI or DMEM	n/a
Mouse bone marrow stem cells	200,000	DMEM	(Gotoh et al., 2020)
Mouse bone marrow triple negative cells	200,000	DMEM	(Gotoh et al., 2020)
Mouse bone marrow stroma cells	10,000	DMEM	n/a
Mouse hepatocyte	40,000	DMEM	n/a

## Key resources table

**Table:** 

REAGENT or RESOURCE	SOURCE	IDENTIFIER
**Bone marrow cell isolation**		

Phosphate-buffered saline (PBS)	Thermo Fisher Scientific	Cat# 10010023
60 mm TC-treated Cell Culture Dish	BD Biosciences	Cat# 353002
15 mL Polystyrene Centrifuge Tube	BD Biosciences	Cat# 352095
50 mL High Clarity PP Centrifuge Tube	BD Biosciences	Cat# 352098
70 μm Cell Strainer	BD Biosciences	Cat# 352350

**RBC lysis buffer**		

Ammonium chloride (NH_4_Cl)	Merck (Sigma-Aldrich)	Cat# A9434-500G
Trizma hydrochloride (Tris)	Merck (Sigma-Aldrich)	Cat# T5941-500G

**mDC culture medium**		

mGM-CSF	PeproTech	Cat# 315-03
RPMI 1640	Merck (Sigma-Aldrich)	Cat# R8758
Penicillin streptomycin	Thermo Fisher Scientific	Cat# 15140122
L-Glutamine	Thermo Fisher Scientific	Cat# 25030081
Non-essential amino acids	Thermo Fisher Scientific	Cat# 11140076
Sodium pyruvate	Thermo Fisher Scientific	Cat# 11360070
Fetal bovine serum (FBS)	Merck (Sigma-Aldrich)	Cat# F0392
2-Mercaptoethanol	Wako	Cat# 137-06862

**Purification of myeloid dendritic cell**		

CD11c MicroBeads UltraPure, mouse	Miltenyi Biotec	Cat# 130-125-835
BSA stock solution	Miltenyi Biotec	Cat# 130-091-376
Rinsing solution	Miltenyi Biotec	Cat# 130-091-222
LS columns	Miltenyi Biotec	Cat# 130-042-401
CD11c antibody	Miltenyi Biotec	Cat# 130-102-800

**TLR ligand**		

LPS	Merck (Sigma-Aldrich)	Cat# L4524
Pam3CSK4	InvivoGen	Cat# tlrl-pms
Poly(I:C)	InvivoGen	Cat# tlrl-pic
Imiquimod (R837)	InvivoGen	Cat# tlrl-imq
CpG-B oligonucleotide; tccatgacgttcctgatgct (uppercase and lowercase letters indicate bases with phosphodiester- and phosphorothioate-modified backbones)	Hokkaido System Science	n/a
ODN 1668	InvivoGen	Cat# tlrl-1668

**Seahorse assays medium**		

RPMI-1640 medium, without glucose and sodium bicarbonate	Merck (Sigma-Aldrich)	Cat# R1383
DMEM without glucose, L-glutamine, phenol red, sodium pyruvate and sodium bicarbonate	Merck (Sigma-Aldrich)	Cat# D5030
D-(+)-Glucose	Merck (Sigma-Aldrich)	Cat# G7021
Sodium pyruvate	Thermo Fisher Scientific	Cat# 11360070
XF RPMI assay medium pack, pH 7.4	Agilent Technologies	Cat# 103681-100
XF RPMI assay medium, pH 7.4	Agilent Technologies	Cat# 103576-100
XF DMEM assay medium pack, pH 7.4	Agilent Technologies	Cat# 103680-100
XF 1.0 M glucose solution	Agilent Technologies	Cat# 103577-100
XF 100 mM pyruvate solution	Agilent Technologies	Cat# 103578-100
XF 200 mM glutamine solution	Agilent Technologies	Cat# 103579-100

**Seahorse assays**		

Seahorse XF24 FluxPak	Agilent Technologies	Cat# 100850-001
Poly-L-lysine solution 0.01%	Merck (Sigma-Aldrich)	Cat# P4707
Poly-D-lysine	Merck (Sigma-Aldrich)	Cat# P7280
Seahorse XFe96 PDL Cell Culture Plates, 6 assays	Agilent Technologies	Cat# 103730-100
Seahorse XFp PDL Cell Culture Miniplates	Agilent Technologies	Cat# 103722-100
FCCP	Merck (Sigma-Aldrich)	Cat# C2920
Rotenone	Merck (Sigma-Aldrich)	Cat# R8875
Antimycin	Merck (Sigma-Aldrich)	Cat# A8674
Oligomycin	Merck (Sigma-Aldrich)	Cat# O4876
Dimethyl sulfoxide (DMSO)	Merck (Sigma-Aldrich)	Cat# D8418
Sodium palmitate	Merck (Sigma-Aldrich)	Cat# P9767
Bovine serum albumin	Merck (Sigma-Aldrich)	Cat# A7030
Etomoxir	Merck (Sigma-Aldrich)	Cat# E1905
2-Deoxy-D-glucose	Merck (Sigma-Aldrich)	Cat# D8375
Sodium palmitate	Merck (Sigma-Aldrich)	Cat# P9767
Bovine serum albumin fraction V	Merck (Sigma-Aldrich)	Cat# 3117057001
NaCl	Merck (Sigma-Aldrich)	Cat# S5886
Seahorse XF Cell Mito Stress Test Kit	Agilent Technologies	Cat# 103015-100
Seahorse XF Glycolytic Rate Assay Kit	Agilent Technologies	Cat# 103344-100
XF Palmitate Oxidation Stress Test Kit	Agilent Technologies	Cat# 103693-100

## Materials and equipment

### Buffer and medium preparation

**Timing: 1–2 h**

**Table:** 0.16M NH_4_Cl solution

Reagent	Stock concentration	Final concentration	Volume/weight
Ammonium chloride (NH4Cl)	n/a	0.16M	8.3g
dH2O	n/a	n/a	1000 mL
**Total**	n/a	n/a	**1000 mL**

**Table:** 0.17M Tris pH 7.65

Reagent	Stock concentration	Final concentration	Volume/weight
Trizma hydrochloride (Tris)	n/a	0.17M	20.6g
dH2O	n/a	n/a	1000 mL
**Total**	n/a	n/a	**1000 mL**

**Table:** RBC lysis solution (pH 7.2)

Reagent	Stock concentration	Final concentration	Volume/weight
0.16M NH4Cl solution	0.16M	0.14M	90 mL
0.17M Tris pH 7.65	0.17M	0.017M	10 mL
**Total**	n/a	n/a	**100 mL**

Preparation of RBC lysis solution

• 8.3 g of Ammonium chloride (NH4Cl) are dissolved. Add dH2O to bring the total volume to 1000 mL.
• 20.6 g of Trizma hydrochloride (Tris) are dissolved. Add dH2O to bring the total volume to 1000 mL Adjust the pH with HCl and NaCl solutions to pH 7.65.
• Mix 90 mL of 0.16M NH4Cl solution and 10 mL of 0.17M Tris pH 7.65. Adjust the pH with HCl and NaCl solutions to pH 7.2. Sterilize RBC lysis solution with Bottle Tops and Filter Units and store at 2°C–8°C.

**Table:** RPMI medium (culture)

Reagent	Stock concentration	Final concentration	Volume/weight
RPMI	n/a	n/a	430 mL
Fetal Bovine Serum	n/a	10% (v/v)	50 mL
Penicillin Streptomycin	10,000 U/mL	1% (v/v)	5 mL
L-glutamine	29.2 mg/mL	1% (v/v)	5 mL
non-essential amino acids	100×	1% (v/v)	5 mL
sodium pyruvate	100 mM	1% (v/v)	5 mL
2-mercaptoethanol	50 mM	50 μM	0.5 mL
**Total**	n/a	n/a	**500 mL**

**Table:** MACS buffer (purification of myeloid dendritic cell)

Reagent	Stock concentration	Final concentration	Volume/weight
Rinsing Solution	n/a	n/a	190 mL
BSA Stock Solution	n/a	n/a	10 mL
**Total**	n/a	n/a	**200 mL**

**Table:** Agilent protocol XF assay medium (mitochondrial stress test)

Reagent	Stock concentration	Final concentration	Volume/weight
XF RPMI assay medium, pH 7.4	n/a	n/a	97 mL
XF 1.0 M Glucose Solution	1000 mM	10 mM	1 mL
XF 100 mM Pyruvate Solution	100 mM	1 mM	1 mL
XF 200 mM Glutamine Solution	200 mM	2 mM	1 mL
**Total**	n/a	n/a	**100 mL**

***Note:*** Please check the latest information on the Agilent website (https://www.agilent.com/en/product/cell-analysis/real-time-cell-metabolic-analysis) before your experiments. We show the protocol of RPMI medium (https://www.agilent.com/cs/library/usermanuals/public/XF24_DAY_OF_MEDIA_PREP.pdf).

**Table:** XF assay medium (glycolysis stress test)

Reagent	Stock concentration	Final concentration	Volume/weight
XF RPMI assay medium, pH 7.4	n/a	n/a	98 mL
XF 100 mM Pyruvate Solution	100 mM	1 mM	1 mL
XF 200 mM Glutamine Solution	200 mM	2 mM	1 mL
**Total**	n/a	n/a	**100 mL**

Preparation of the XF assay medium (glycolysis stress test)

• Warm the assay medium to 37°C without CO2.
• Adjust the pH with HCl and NaCl solutions to pH 7.4. Filter using polycarbonate membrane filter.
• Keep at 37°C until ready to use without CO2.

**CRITICAL:** The value of ECAR is affected by the pH of assay medium. Therefore, to accurately measure the ECAR value, it is necessary to adjust the pH to 7.4 4 h before assay under 37°C.

***Note:*** Please check the latest information on the Agilent website (https://www.agilent.com/en/product/cell-analysis/real-time-cell-metabolic-analysis) before your experiments. We show the protocol of RPMI medium (https://www.chem.agilent.com/cs/library/usermanuals/public/XF_Glycolysis_Stress_Test_Kit_User_Guide.pdf).

**Table:** XF assay medium (palmitate oxidation stress test)

Reagent	Stock concentration	Final concentration	Volume/weight
XF RPMI assay medium, pH 7.4	n/a	n/a	98 mL
XF 1.0 M Glucose Solution	1000 mM	10 mM	1 mL
XF 200 mM Glutamine Solution	200 mM	2 mM	1 mL
**Total**	n/a	n/a	**100 mL**

***Note:*** Please check the latest information on the Agilent website (https://www.agilent.com/en/product/cell-analysis/real-time-cell-metabolic-analysis) before your experiments. We show the protocol of RPMI medium (https://www.agilent.com/cs/library/flyers/public/flyer-agilent-seahorse-xf-palmitate-oxidation-stress-test-kit-cell-analysis-5994-1649en-agilent.pdf).

**Table:** Our protocol XF assay medium (TLR-induced metabolic assay, mitochondrial stress test)

Reagent	Stock concentration	Final concentration	Volume/weight
RPMI-1640 Medium, without glucose and sodium bicarbonate with L-Glutamine	n/a	n/a	8.4 g
Fetal Bovine Serum (FBS)	n/a	10% (v/v)	100 mL
sodium pyruvate	100 mM	1% (v/v)	10 mL
D-(+)-Glucose	n/a	1000 mg/L (w/v)	1000 mg
dH2O	n/a	n/a	890 mL
**Total**	n/a	n/a	**1000 mL**

***Note:*** We recommend using within 4 h of medium preparation to avoid reagents degradation over the time and pH changes. In addition, we recommend warming the assay medium in a 37°C incubator without CO2 1 h before its use on cells.

***Note:*** Because FBS contains a high amount of different unknown components, the amount of FBS may affect the result of ECAR and OCR**.** Depending on the results of ECAR and OCR, it should be considered to reduce the amount of FBS.

**Table:** XF assay medium (Glycolysis stress test)

Reagent	Stock concentration	Final concentration	Volume/weight
RPMI-1640 Medium, without glucose and sodium bicarbonate	n/a	n/a	8.4 g
Fetal Bovine Serum	n/a	10% (v/v)	100 mL
sodium pyruvate	100 mM	1% (v/v)	10 mL
dH2O	n/a	n/a	890 mL
**Total**	n/a	n/a	**1000 mL**

**Table:** XF assay medium (palmitate oxidation stress test)

Reagent	Stock concentration	Final concentration	Volume/weight
RPMI-1640 Medium, without glucose and sodium bicarbonate	n/a	n/a	8.4 g
sodium pyruvate	100 mM	1% (v/v)	10 mL
D-(+)-Glucose	n/a	1000 mg/L (w/v)	1000 mg
dH2O	n/a	n/a	890 mL
**Total**	n/a	n/a	**1000 mL**

Preparation of the XF assay medium

• Once 8.4 g of RPMI-1640 medium powder, D-glucose, fetal bovine serum, and sodium pyruvate are dissolved, add dH20 to bring the total volume to 1000 mL.
• Adjust the pH with HCl and NaCl solutions to pH 7.4.
• Sterilize the cell culture media with Bottle Tops and Filter Units and store at 2°C–8°C.

**CRITICAL:** Adjust pH as precisely as possible; the final pH will affect the results of ECAR data. Because glycolysis is measured by the changes in extracellular pH, the XF assay medium should not contain any buffering reagents.

***Note:*** If you use other cells, prepare a DMEM-based medium by referring to Table 1.

**Table:** Oligomycin stock

Reagent	Stock concentration	Final concentration	Volume/weight
Oligomycin	n/a	10 mM	5 mg
DMSO	n/a	n/a	632 μL
**Total**	n/a	n/a	**632 μL**

**Table:** FCCP stock

Reagent	Stock concentration	Final concentration	Volume/weight
FCCP	n/a	100 mM	10 mg
DMSO	n/a	n/a	393 μL
**Total**	n/a	n/a	**393 μL**

**Table:** Rotenone stock

Reagent	Stock concentration	Final concentration	Volume/weight
Rotenone	n/a	10 mM	5 mg
DMSO	n/a	n/a	1267 μL
**Total**	n/a	n/a	**1267 μL**

**Table:** Antimycin stock

Reagent	Stock concentration	Final concentration	Volume/weight
Antimycin	n/a	10 mM	5 mg
DMSO	n/a	n/a	393 μL
**Total**	n/a	n/a	**393 μL**

### Preparation of palmitate (PA) + bovine serum albumin (BSA) conjugate

**Timing: 1–2 h**

***Note:*** We recommend using the XF Palmitate Oxidation Stress Test Kit (Cat# 103693-100)

**Figure 1:**
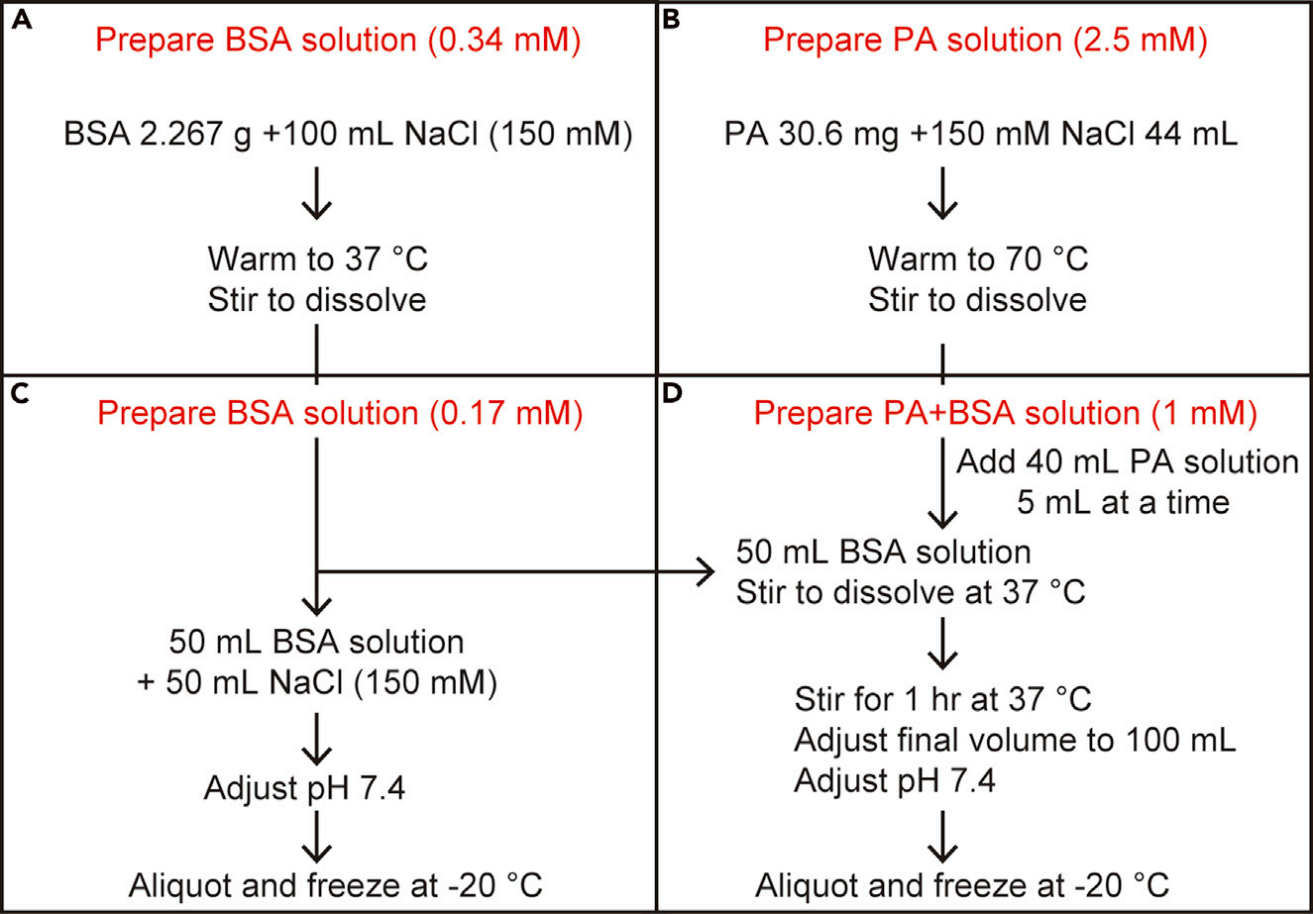
Preparation of palmitate (PA) + bovine serum albumin (BSA) conjugate

If you prepare PA+BSA yourself, refer Figure 1 or http://www.wklab.org/wp-content/uploads/2016/02/Palmitate-BSA_Prep_SOP_v080624.pdf.

**Table:** BSA solution

Reagent	Stock concentration	Final concentration	Volume/weight
Bovine Serum Albumin Fraction V	n/a	0.34 mM	2.267 g
150 mM NaCl	150 mM	150 mM	100 mL
**Total**	n/a	n/a	**100 mL**

Preparation of the BSA solution (Figure 1A)

• Add 100 mL of 150 mM NaCl in a 250 mL glass beaker. Warm the beaker with a stir bar in a 37°C incubator.
• Add 2.267 g of BSA to 100 mL of 150 mM NaCl in a 250 mL glass beaker. Stir until the BSA is completely dissolved.

**Table:** PA solution

Reagent	Stock concentration	Final concentration	Volume/weight
Sodium palmitate (PA)	n/a	2.5 mM	30.6 mg
150 mM NaCl	150 mM	150 mM	44 mL
**Total**	n/a	n/a	**44 mL**

Preparation of the PA solution (Figure 1B)

• Add 44 mL of 150 mM NaCl to a 100 mL glass beaker. Warm the beaker with a stir bar in a 70°C incubator.
• Add 30.6 mg PA to 44 mL of 150 mM NaCl in a 100 mL glass beaker. The PA solution may appear increasingly cloudy as the temperature reaches 50–60°C but will clarify approaching 70°C. Stir until the PA is completely dissolved.

**CRITICAL:** PA is difficult to dissolve at temperatures of <50°C. Therefore, take care not to lower the PA solution's temperature to <50°C until PA binds to BSA. If PA do not conjugate to BSA, PA is not incorporated into cells. Therefore, this section is important.

**Table:** PA+BSA solution

Reagent	Stock concentration	Final concentration	Volume/weight
PA solution	2.5 mM (PA)	1 mM (PA)	40 mL
BSA solution	0.34 mM (BSA)	0.17 mM (BSA)	50 mL
150 mM NaCl	150 mM	150 mM	10 mL
**Total**	n/a	n/a	**100 mL**

Conjugating PA to BSA (Figure 1D)

**CRITICAL:** If PA do not conjugate to BSA, PA is not incorporated in DCs. Therefore, this section is important. In particular, because PA is difficult to dissolve at temperatures of <50°C, be careful the temperature of PA solution.

• Add 50 mL of 150 mM NaCl to a 250 mL glass beaker. Warm the beaker with a stir bar in a 37°C incubator. Transfer 5 mL of the PA solution with a 10 mL pipette into a 250 mL glass beaker.
• Add total of 40 mL of PA solution into 250 mL glass beaker in 8 batches of 5 mL each. Stir a 250 mL glass beaker at 37°C for 1 h.
• Add 10 mL of 150 mM NaCl to a 250 mL glass beaker and adjust the final volume to 100 mL.
• Check the solution’s pH and adjust it to pH 7.4.
• Separate into 2–5 mL each and freeze at −20°C.

## Step-by-step method details

### Bone marrow cell isolation

**Timing: 3 h**

***Note:*** Ensure that all the reagents and samples are kept on ice during the entire procedure. Perform all steps in a laminar flow hood with sterile equipment to maintain sterility.

For 2–4 whole Seahorse XF24 cell culture microplates, you will need BMDCs from one mouse.

***Note:*** We recommend using male 6–10 week-old mice.

1. Sterilize the dissection kit and bench area with 70% (vol/vol) ethanol spray. Euthanize the mice with CO_2_ or inhaled anesthetics, such as isoflurane or sevoflurane, followed by cervical dislocation.
2. Pin down the mouse to expose its abdomen. Spray the euthanized mice with 70% (vol/vol) ethanol to sterilize its skin.
3. Use scissors to cut along the midline of the abdomen until exposing the femurs. Remove the femurs and tibias and place them in a dish containing PBS. (Methods video S1)

4. Remove the muscle and as much connective tissue as possible from the femurs and tibias. Place the harvested bones on a 60-mm dish filled with PBS.

**CRITICAL:** If the bone is broken or the soft tissue is inadequately removed, the number of bone marrow cells harvested may be decreased.

Methods video S1. Collect femurs and tibias from mouse (step 3)

This section is shown in Methods video S2.

5. Trim both ends of the femurs and tibias carefully using sterile scissors to expose the interior marrow shaft (Methods video S2).
6. Use a 5 mL syringe to draw up to 5 mL of fresh culture medium. Attach a 21-gauge needle to the syringe. Hold the bone over a fresh 60-mm dish, with its narrow end pointing down. Flush the marrow out of the bone with 5 mL of PBS (Methods video S3).

Methods video S2. Trim both ends of the femurs and tibias (step 5)

7. Aspirate the marrow and the medium from the 60-mm dish and pipette up and down three times, rinsing the sides of the dish each time to disperse the marrow.
8. Collect the cell suspension with the syringe and add the suspension to a 15 or 50 mL tube.

***Optional:*** Pass the cell suspension through a 70-μm cell strainer to remove any remaining bone or muscle fragments.

9. Centrifuge the cell suspension at 3,000× rpm (800 × *g*) at 4°C for 5 min to pellet the cells. Discard the supernatant by gently tilting the tube and pouring the media into a waste disposal beaker. Recap the tube and gently tap to disperse the cell pellet.
10. Add 1.0 mL / body of RBC lysis buffer to the cells and gently tap the tube with fingers to mix the lysis solution for 30–60 s. Add 10 mL of RPMI medium to dilute the buffer after RBC lysis.
11. Centrifuge the cell suspension at 3,000× rpm (800 × *g*) at 4°C for 5 min to pellet the cells. Discard the supernatant by gently tilting the tube and pouring the media into a waste disposal beaker. Recap the tube and gently tap to disperse the cell pellet.

**CRITICAL:** If red blood cell lysis fails, return to step 10.

12. Resuspend the cells in 10 mL of culture medium and place on ice. Mix the cell culture medium well and count the cells; 2–6 × 10^7^ bone marrow cells can be collected from two tibias and two femurs.
13. Day 0: Seed 2 × 10^6^ bone marrow cells in 2 mL of culture medium onto a 12-well culture plate with 10–25 ng/mL of GM-CSF in a 37°C and humidified 5% CO_2_ incubator.

***Note:*** BMDCs can be cultured in a 60-mm dish (12 × 10^6^ bone marrow cells/ 6 mL), a 100-mm dish (2 × 10^7^ bone marrow cells/ 10 mL), or a 6-well dish (8 × 10^6^ bone marrow cells/ 4 mL).

Methods video S3. Collect bone marrow cell suspension (steps 6–8)

If you wish to culture plasmacytoid dendritic cells (pDCs) or macrophages, you can obtain pDCs and macrophages using Flt3 ligands or M-CSF at the same concentration as GM-CSF (Gotoh et al., 2008) (Sasaki et al., 2017).

### Myeloid dendritic cell culture

**Timing: 6–7 days**

14. Day 3, 5: Add 2 mL of culture media with 10–25 ng/mL GM-CSF and divide 1 well into 2 wells.
15. Day 6–7: Collect the myeloid dendritic cells. We speculate that 1–3 × 10^7^ DCs / body can be recovered.

**CRITICAL:** Be careful to avoid collecting the highly adherent cells.

Important: Non-BMDCs can be eliminated by early washing steps, discarding highly adherent cells, and enriching or sorting for CD11c^+^ cells (Helft et al., 2015).

***Note:*** If the culture media is not added properly, the survival rate and number of the BMDCs will be reduced.

### Purification of myeloid dendritic cell

**Timing: 2 h**

***Note:*** The percentage of CD11c^+^ BMDCs is approximately 60%–90%. Use the following process to obtain a more concentrated population of CD11c^+^ BMDCs. After this step, we speculate that 0.5–1 × 10^7^ DCs / body can be recovered.

***Note:*** If you will purify CD11c^+^ BMDCs, refer https://www.miltenyibiotec.com/DE-en/shop/comMiltenyiDatasheet/product?productId=54985.

16. Mix the cell culture medium well and count the cells.
17. Centrifuge the cell suspension at 3,000× rpm (800 × *g*) at 4°C for 5 min to pellet the cells. Discard the supernatant by gently tilting the tube and pouring the media into a waste disposal beaker. Recap the tube and gently tap to disperse the cell pellet.
18. Resuspend the cell pellet in 10 mL of MACS buffer.
19. Centrifuge the cell suspension at 3,000× rpm (800 × *g*) at 4°C for 5 min. Discard the supernatant by gently tilting the tube and pouring the media into a waste disposal beaker. Recap the tube and gently tap to break up the cell pellet.
20. Resuspend the cell pellet in 400 μL of MACS buffer per 1–2 × 10⁸ cells.
21. Add 100 μL of CD11c MicroBeads UltraPure per 1– 2 × 10⁸ total cells.
22. Mix well and incubate for 10 min in the dark in the refrigerator at 2°C–8°C.
23. After incubation, add 10 mL of MACS buffer and centrifuge the cell suspension at 3,000× rpm (800 × *g*) at 4°C for 5 min. Discard the supernatant. Recap the tube and gently tap to disperse the cell pellet.
24. Resuspend cell pellet in 1 mL of MACS buffer.
25. Place an LS column in the magnetic field of the MACS Separator. Add 3 mL of MACS buffer onto the LS column to rinse the column.
26. Apply 1 mL of the cell suspension onto the column. After adding the cell suspension, wash the LS column three times with 3 mL MACS buffer each.
27. Remove the LC column from the MACS Separator and place it on a 15 mL collection tube.
28. Apply 5 mL of MACS buffer onto the column. Immediately flush out the CD11c^high^ mDCs by firmly pushing the plunger into the column.

***Optional:*** If you use the autoMACS® Pro Separator, refer to the corresponding user’s manual on using the autoMACS® Pro Separator.

***Note:*** After collecting the CD11c^high^ mDCs, we recommend checking the purity of BMDCs by flow cytometry. If you will check the purity of BMDCs, refer https://www.biolegend.com/en-us/protocols/cell-surface-flow-cytometry-staining-protocol.

### Hydration of a Seahorse XFe sensor cartridge

29. Place the assay cartridge upside down next to a 24-well utility plate. Add 1.0 mL of XF calibrant solution to each well of the 24-well utility plate.
30. Put the cartridge back onto the utility plate and the sensor cartridge with the lid.
31. Place it in a 37°C incubator without CO_2_ to hydrate for 4–48 h. To prevent evaporation of the water, verify that the incubator is properly humidified.

***Note:*** The sensor cartridge should be hydrated for at least 4 h before assay. However, we do not recommend using an XFe sensor cartridge after >48 h of hydration.

### Preparation of poly-L-lysine-coated microplates

***Note:*** If you use non-adherent cells including BMDCs, we recommend this step. The adhesion of cells to a plate affects the accuracy of the experiment.

32. Apply 50 μL of 0.01% poly-L-Lysine (PLL) or poly-D-Lysine (PDL) to the wells of the 24-well XF cell culture microplate. Tap the microplate to ensure that the liquid completely covers the bottom of the well.
33. Incubate the microplate with the PLL or PDL solution for at least 5 min at room temperature.
34. Remove the PLL or PDL solution by aspiration and thoroughly rinse the bottom of the plate with sterile water. Dry for at least 2 h before seeding cells.

***Note:*** If you use PLL, please check this information on the Sigma Aldrich website (https://www.sigmaaldrich.com/technical-documents/articles/biofiles/poly-lysine-product.html). If you use PDL, please check this information on the Sigma Aldrich website (https://www.sigmaaldrich.com/catalog/product/sigma/p7280?lang=en&region=US). If you use Cell Tak, please check this information on the Agilent Technologies website (https://www.agilent.com/cs/library/technicaloverviews/public/5991-7153EN.pdf).

***Note:*** If you use PDL-coated cell culture microplate, you can get PDL-coated cell culture microplate (#103730-100, #103722-100) from Agilent Technologies. (https://www.agilent.com/cs/library/flyers/public/flyer-agilent-seahorse-xf-pdl-coated-cell-culture-microplates-cell-analysis-5994-1990en-agilent.pdf)

### Seeding mDCs in XF24 culture microplate

35. Collect the BMDCs into a 15 or 50 mL Falcon tube and count the cells.
36. Centrifuge the cell suspension at 3,000× rpm (800 × *g*) at 4°C for 5 min to pellet the cells. Discard the supernatant by gently tilting the tube and pouring the media into a waste disposal beaker. Recap the tube and gently tap to disperse the cell pellet.
37. Resuspend the cell pellet in XF assay medium at a density of approximately 2 × 10^6^ /mL. Mix the cell suspension well and seed 100 μL of it on a PLL-coated 24-well XF microplate without a blank well.

***Note:*** This protocol describes measuring oxygen consumption rates (OCR) and ECAR at 2 × 10^5^ BMDCs/well using an XF24 analyzer. If you would like to analyze only OCR, you will obtain the results by reducing the number of mDCs used. If you would like to analyze splenic dendritic cells or plasmacytoid dendritic cells, you should seed 2–5 × 10^5^ mDCs/well.

38. Centrifuge the microplate for 2–5 min at 2000 rpm at room temperature to allow the cells to settle into a monolayer at the bottom of the plate.

***Note:*** BMDCs are non-adherent cells. Therefore, to use swinging buckets or plates attach BMDCs to the bottom of the plate.

**Figure 2:**
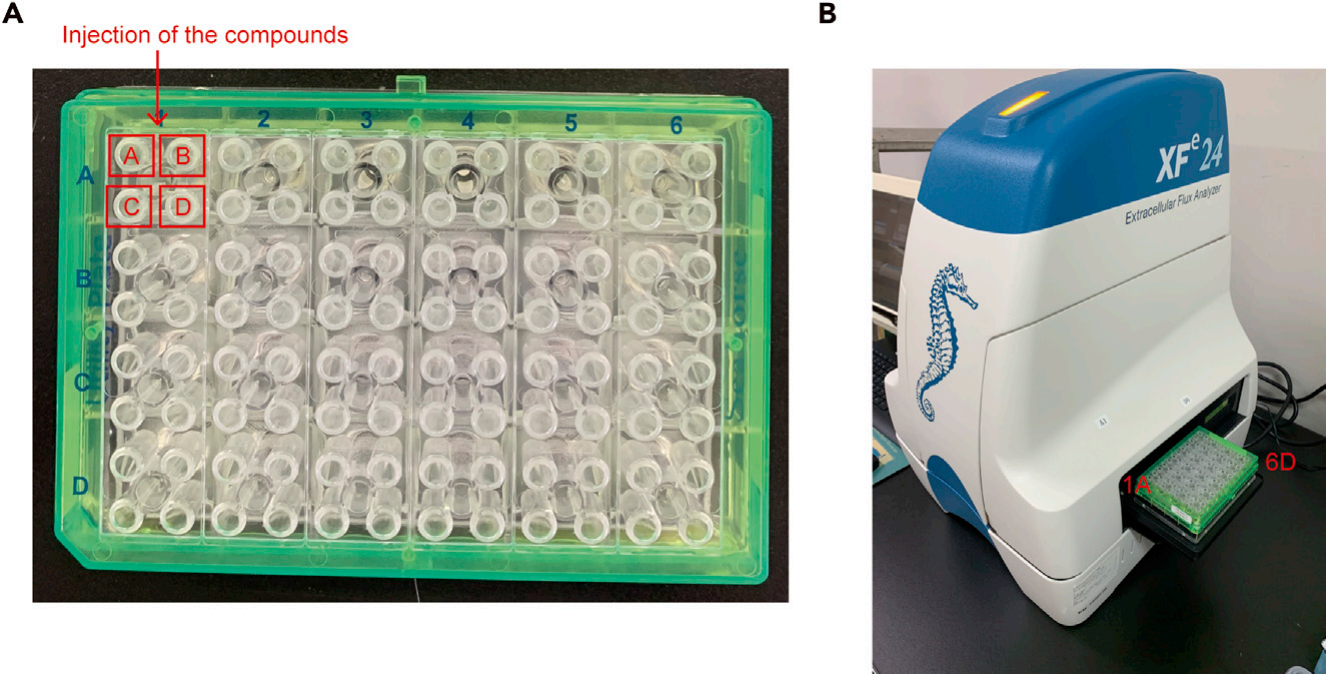
Photographs of XFe sensor cartridge and XF24 extracellular flux analyzer

39. Slowly add 400 μL of XF assay medium into the sample to avoid disrupting the cell monolayer. Add 500 μL of XF assay medium into the blank well. Normally, 4 wells (1A, 3C, 4B, 6D) are used as a blank well (Figure 2B).

**CRITICAL:** No cells should be placed in the blank wells.

40. Incubate the microplate for 30–60 min at 37°C without CO_2_ until you are ready to load the plate onto the XF24 extracellular flux analyzer.

**CRITICAL:** This session is critical for obtaining accurate results of the Seahorse assay. The number of cells directly affects the values of OCR and ECAR. If you seed too many cells, the cells will be seeded in multiple layers. You should observe the cells under an inverted microscope to confirm that a monolayer of cells is present in all the wells.

### XF assay

**Figure 3:**
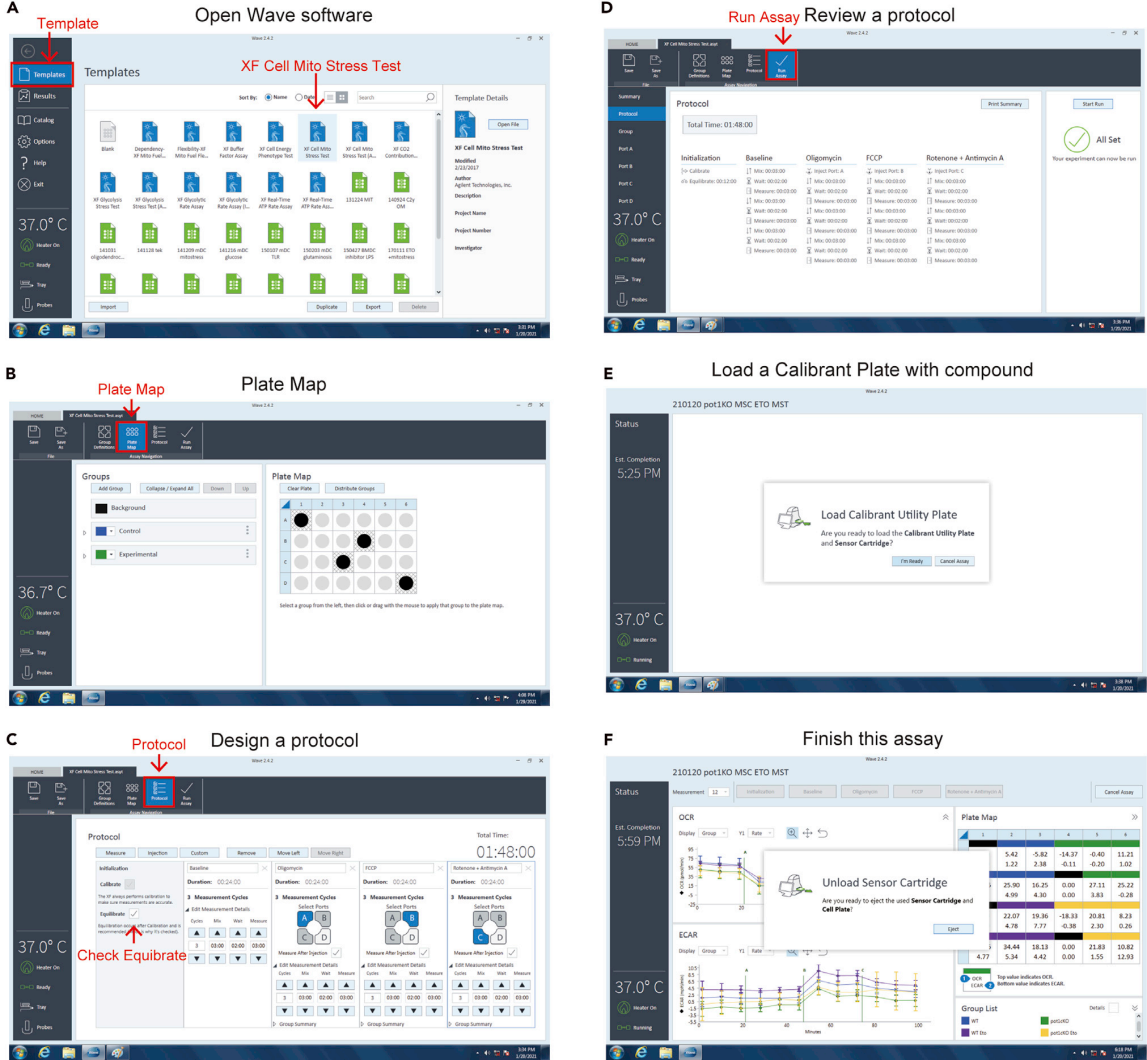
Management of the assay protocol (A) Open software and select a protocol. (B) Select Blank and sample wells. (C) Design a protocol. (D) Check a protocol. (E) Load a calibrant plate with compounds. (F) Finish this assay.

41. Prepare 2 mL of 10× TLR agonist, mitochondrial inhibitor, or uncoupler injection solutions and an XFe sensor cartridge.
42. Add 56–75 μL of each 10× injection solution into injection ports A, B, C, and D, respectively (Figure 2A). Return the hydration cartridge to the 37°C incubator without CO_2_ before setting up the run.
43. Design a study protocol in the XF24 extracellular flux analyzer software provided by the manufacturer (Figures 3A–3D). Click the “I’m Ready” button and place the hydrated cartridge from the 37°C incubator without CO_2_ on the XF24 extracellular flux analyzer (Figures 2B and 3E).

**Figure 4:**
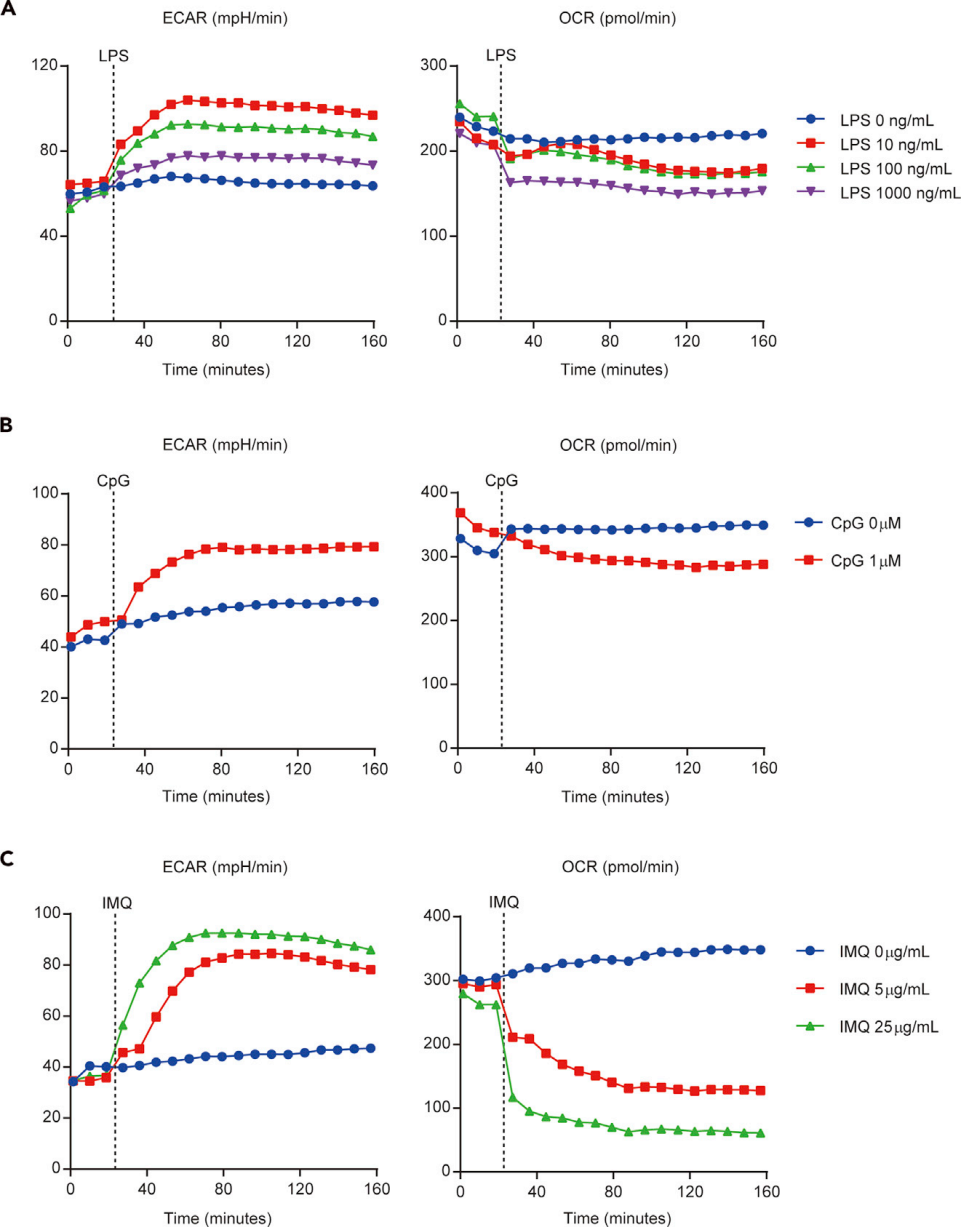
Metabolic change by TLR stimulation Real-time changes in the ECAR and OCR of BMDCs treated with LPS (A,B), CpG (C, D), and IMQ (E, F).

**Figure 5:**
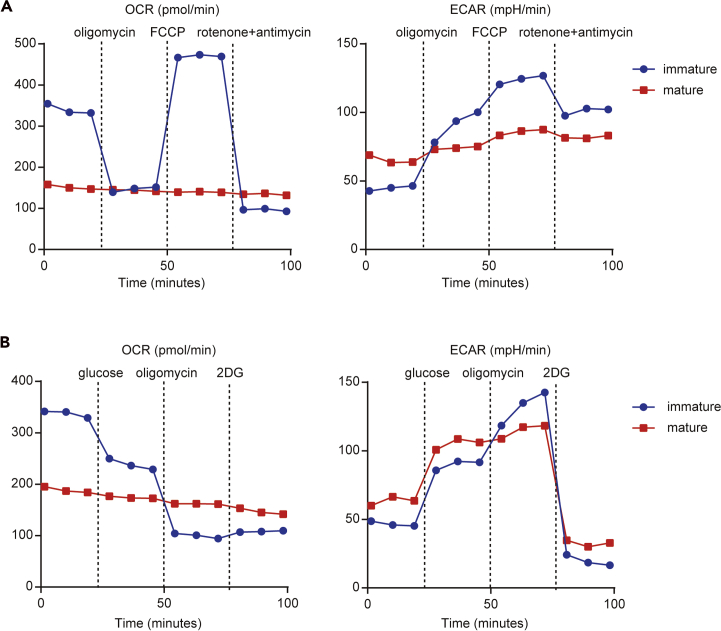
ECAR and OCR of immature and mature BMDCs BMDCs (2 × 10^5^ cells/well) stimulated without (immature) or with (mature) LPS (100 ng/mL) for 12 h were seeded in an XF-24 extracellular flux analyzer. The real-time OCR and ECAR were measured during the sequential treatments with a mitochondrial stress test (A: oligomycin, FCCP, antimycin-A/rotenone) and glycolysis stress test (B: Glucose, oligomycin, 2-Deoxy-D-glucose (2DG)).

**Figure 6:**
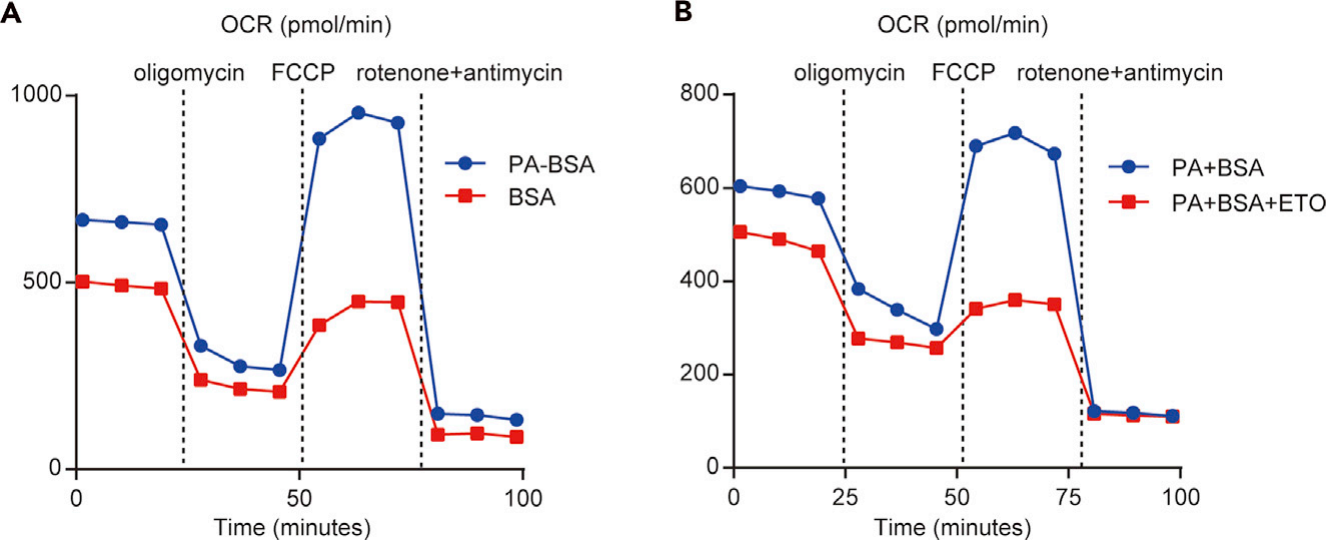
OCR of BMDCs for FAO assay Wild-type DCs (2 × 10^5^ cells/well) pretreated with PA+BSA (A) and Etomoxir (B) for 30 min were seeded in an XF-24 extracellular flux analyzer. The real-time OCR was determined during the sequential treatments with a mitochondrial stress test (A: oligomycin, FCCP, antimycin-A/rotenone).

***Note:*** We recommend 4 blank (Figure 3B) and 3 or more replicas wells for 1 plate.

44. Wait for the machine to calibrate the sensors. When the calibration is finished, keep the cartridge on the machine while the calibrant plate in the load position is sent out. At this point, take the XF24 cell culture microplate from the 37°C incubator without CO_2_ and put it into the load position and click the “CONTINUE” button.
45. After the XF24 extracellular flux analyzer run is finished, remove the assayed XF24 cell culture microplate, place it in a 37°C incubator to determine the cell counts or the protein concentration, and click the “CONTINUE” button to end the program.

### After XF assay (optional)

***Note:*** Normalization is an important component in the workflow for performing analysis of raw data to ensure accurate and consistent interpretation of results. We show a method using the number of cells. If you will use the method of normalization (e.g., using protein concentration, cell count, DNA content), refer https://www.agilent.com/cs/library/technicaloverviews/public/Methods_and_Strategies_for_Normalizing_Tech_Overview_022118.pdf

***Note:*** If XF plate is coated with protein containing cellular adherents (e.g., collagen, laminin, Matrigel), normalization using total protein is also not applicable.

46. Carefully remove the supernatant and add PBS 100 μL.
47. Strip off the cells on bottom and count the cells.
48. Standardize using Wave software.

## Expected outcomes

Measuring TLR-induced metabolic changes: The binding of TLR and TLR agonists leads to rapid activation of DCs (Kawai and Akira, 2011). Activated DCs by TLR agonist, including LPS (TLR4 ligand) and CpG-DNA (TLR9 ligand), exhibit a rapid increase in glucose consumption and lactate production, as indicated by the real-time changes in extracellular acidification (ECAR) and OCR (Everts et al., 2014, Gotoh et al., 2018). DC activation is dependent on an early increase in glycolysis. Conversely, LPS and CpG-DNA do not affect the mitochondrial respiratory chain in the DCs. Imiquimod, a TLR7 agonist, also enhances glycolysis of DCs. Unlike LPS and CpG, imiquimod impairs the mitochondrial respiratory chain by binding and inhibiting the quinone oxidoreductases NQO2 (Gross et al., 2016). Figure 4 depicts ECAR and OCR measurements to detect metabolic changes by TLR stimulation.

Measuring post TLR stimulation changes: Several studies have also shown that immature DCs use mitochondrial oxidative phosphorylation (OXPHOS) as a core metabolic process, rather than glycolysis. Conversely, mature DCs induced by TLR ligands display increased glycolysis and inactivated OXPHOS (Everts et al., 2012, Gotoh et al., 2018). Analysis can be done to measure the difference in mitochondrial and glycolytic stress between the immature and mature DCs (Figure 5).

Measuring fatty acid metabolism in DCs: Fatty acid oxidation has critical roles in regulating adaptive and innate immune responses (O'Neill et al., 2016). Tolerogenic and mature DCs showed substantially different levels of proteins and metabolites involved in the fatty acid oxidation (FAO) pathway (Malinarich et al., 2015) (Figure 6).

The individual protocols to measure each of these metabolic changes are outlined in “quantification and statistical analysis.”

## Quantification and statistical analysis

### Analysis of TLR-induced metabolic change

**Table:** Inject port A (TLR agonist)

Port	Compound	Concentration of port	Add to port volume (μL)	Final concentration
A	LPS	100 ng/mL	56	100 ng/mL
A	LPS	1000 ng/mL	56	100 ng/mL
A	CpG	10 μM	56	1 μM
A	IMQ	50 μg/mL	56	5 μg/mL
A	IMQ	250 μg/mL	56	25 μg/mL

**Table:** Protocol commands

Calibrate		
Equilibrate		
Mix	3 min	Perform 3×
Wait	2 min	
Measure	3 min	
Inject from port A: TLR agonist (LPS, IMQ, CpG, et. al)		
Mix	3 min	Perform 8–12×
Wait	2 min	
Measure	3 min	
End		

### Analysis of metabolic change after TLR stimulation

Pretreatment (DC maturation)

1. Stimulate BMDCs with LPS (100 ng/mL) or CpG-DNA (1 μM) for at least 12 h in a 37°C and humidified 5% CO2 incubator.
2. Collect the BMDCs into a 15- or 50-mL Falcon tube and count the cells.
3. Centrifuge the cell suspension at 3,000× rpm at 4°C for 5 min to pellet the cells. Discard the supernatant by gently tilting the tube and pouring the media into a waste disposal beaker. Recap the tube and gently tap to disperse the cell pellet.
4. Resuspend the cell pellet in XF assay medium at a density of approximately 2 × 10^6^ / mL. Mix the cell suspension and seed 100 μL of it on a PLL-coated 24-well XF microplate without a blank well.
5. Centrifuge the microplate for 2–5 min at 2000 rpm at room temperature to allow the cells to settle into a monolayer at the bottom of the plate. Proceed by same way as for Method 37.
6. Slowly add 400 μL of XF assay medium into the sample to avoid disrupting the cell monolayer. Add 500 μL of XF assay medium into the blank well.
7. Incubate the microplate for 30–60 min at 37°C without CO2 until you are ready to load the plate onto the XF24 extracellular flux analyzer.

**Table:** Inject port (mitochondrial stress test)

Port	Compound	Concentration of port (μM)	Add to port volume (μL)	Final concentration (μM)
**A**	Oligomycin	2.5	56	0.25
**B**	FCCP	40	62	4
**C**	Rotenone+Antimycin	10	68	1

**Table:** Inject port (glycolysis stress test)

Port	Compound	Concentration of port	Add to port volume (μL)	Final concentration
**A**	Glucose	100 mM	56	10 mM
**B**	Oligomycin	2.5 μM	62	0.25 μM
**C**	2-DG	500 mM	68	50 mM

***Note:*** Because the ideal compound concentration is affected by cell type and assay medium, we recommend that a titration experiment for these compounds is performed for new cells or assay medium. If you check the ideal compound concentration of mitochondrial stress test, refer the Agilent website in p14–15 (https://www.agilent.com/cs/library/usermanuals/public/XF_Cell_Mito_Stress_Test_Kit_User_Guide.pdf).

**Table:** Protocol commands (mitochondrial stress test/glycolysis stress test)

Calibrate		
Equilibrate		
Mix	3 min	Perform 3×
Wait	2 min	
Measure	3 min	
Inject from port A: oligomycin/glucose		
Mix	3 min	Perform 3×
Wait	2 min	
Measure	3 min	
Inject from port B: FCCP/oligomycin		
Mix	3 min	Perform 3×
Wait	2 min	
Measure	3 min	
Inject from port C: Rotenone+Antimycin/2-DG		
Mix	3 min	Perform 3×
Wait	2 min	
Measure	3 min	
End		

### Analysis of fatty acid metabolism in myeloid dendritic cells

Pretreatment (FAO assay)

1. Collect the BMDCs into a 15 or 50 mL Falcon tube and count the cells.
2. Centrifuge the cell suspension at 3,000× rpm at 4°C for 5 min to pellet the cells. Discard the supernatant by gently tilting the tube and pouring the media into a waste disposal beaker. Recap the tube and gently tap to disperse the cell pellet.
3. Resuspend the cell pellet in XF assay medium at a density of approximately 2 × 10^6^ /mL. Mix the cell suspension well and seed 100 μL of it on a PLL-coated 24-well XF microplate without a blank well.
4. Centrifuge the microplate for 2–5 min at 2000 rpm at room temperature to allow the cells to settle into a monolayer at the bottom of the plate.
5. Slowly add 400 μL of XF assay medium with PA (0.3 mM) +BSA (0.05 mM), BSA (0.05 mM), or 200 μM Etomoxir into the sample to avoid disrupting the cell monolayer. Add 500 μL of XF assay medium into the blank well.
6. Incubate the microplate for 30–60 min at 37°C without CO2 until you are ready to load the plate onto the XF24 extracellular flux analyzer (Figure 6).

**Note:** Recently, several studies showed that Etomoxir induces Cpt1a-independent off-target effects at concentrations >10 or 100 μM (Divakaruni et al., 2018) (Raud et al., 2018). Therefore, if you want to analyze only the CPT1a-specific inhibitory effect, we recommend to analysis with Etomoxir at concentrations < 10 μM.

**Table:** Inject port (mitochondrial stress test)

Port A	Concentration of port A	Final concentration
Oligomycin	2.5 μM	0.25 μM
**Port B**	**Concentration of port B**	**Final concentration**
FCCP	40 μM	4 μM
**Port C**	**Concentration of port C**	**Final concentration**
Rotenone+Antimycin	10 μM	1 μM

**Table:** Protocol commands

Calibrate		
Equilibrate		
Mix	3 min	Perform 3×
Wait	2 min	
Measure	3 min	
Inject from port A: oligomycin/glucose		
Mix	3 min	Perform 3×
Wait	2 min	
Measure	3 min	
Inject from port B: FCCP/oligomycin		
Mix	3 min	Perform 3×
Wait	2 min	
Measure	3 min	
Inject from port C: Rotenone+Antimycin/2-DG		
Mix	3 min	Perform 3×
Wait	2 min	
Measure	3 min	
End		

### XF assay analysis

***Note:*** Agilent Seahorse Wave Desktop software is the assay design, data analysis, and file management software for all Agilent Seahorse XF Analyzers. Because the latest release of Wave Desktop software provides new analysis capabilities in a redesigned interface that will streamline your XF workflow, download from the following site. https://www.agilent.com/en/products/cell-analysis/software-download-for-wave-desktop?productURL=https%3A%2F%2Fwww.agilent.com%2Fen%2Fproduct%2Fcell-analysis%2Freal-time-cell-metabolic-analysis%2Fxf-software%2Fseahorse-wave-desktop-software-740897

1. Open and view assay result file with Agilent Seahorse Wave Desktop software (Figure 7A).

**Figure 7:**
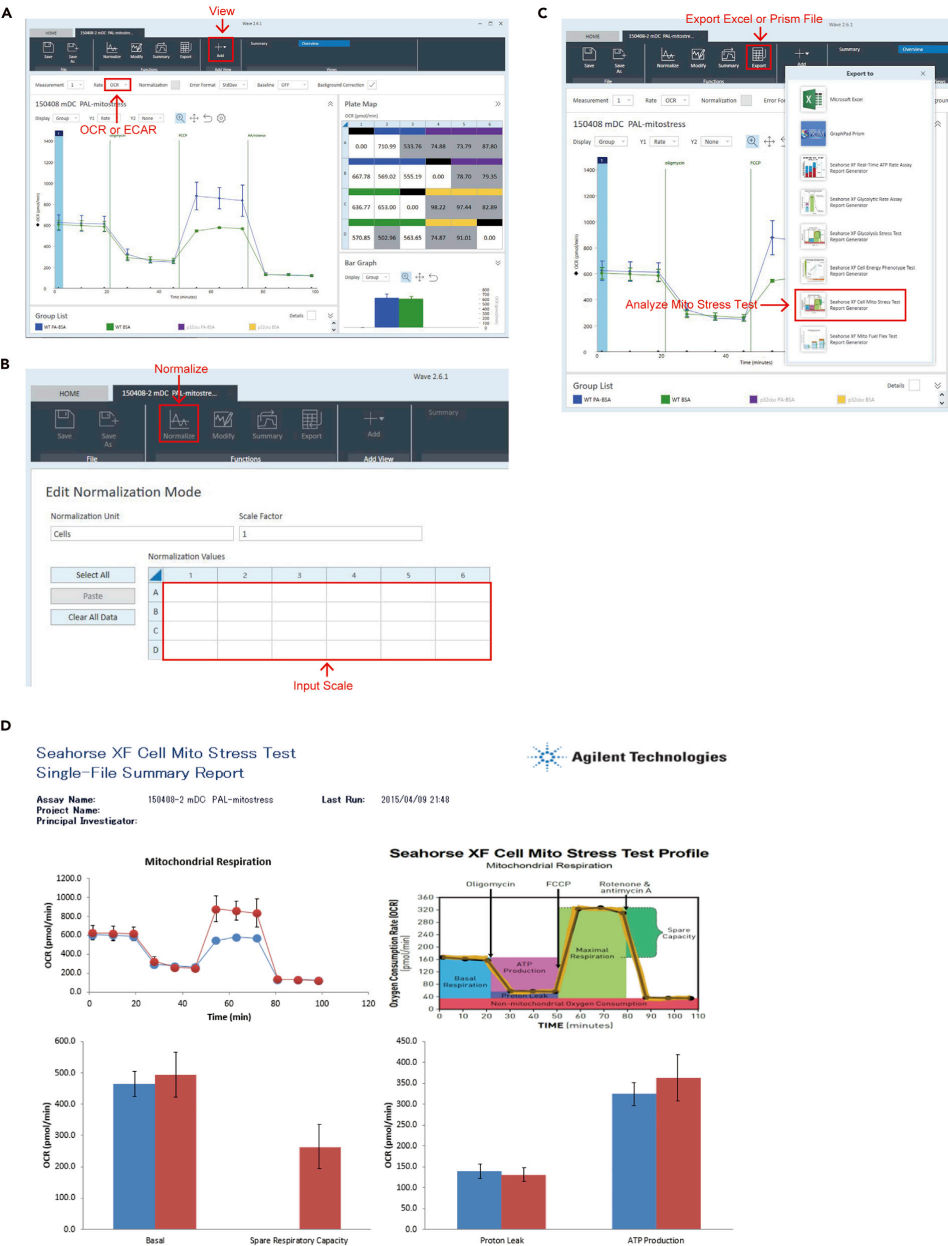
Analysis of XF assay (A) Open software and select OCR or ECAR. (B) Normalization of the assay. (C) Export Excel or Prism. (D) Printscreen of XF Mito Stress Assay.
2. Normalize by the number of cells or the concentration of protein in each well (Figure 7B).
3. Export the assay result as Microsoft Excel or GraphPad Prism file (Figure 7C).
4. Analyze the assay results (Figures 7D and 8).

**Figure 8:**
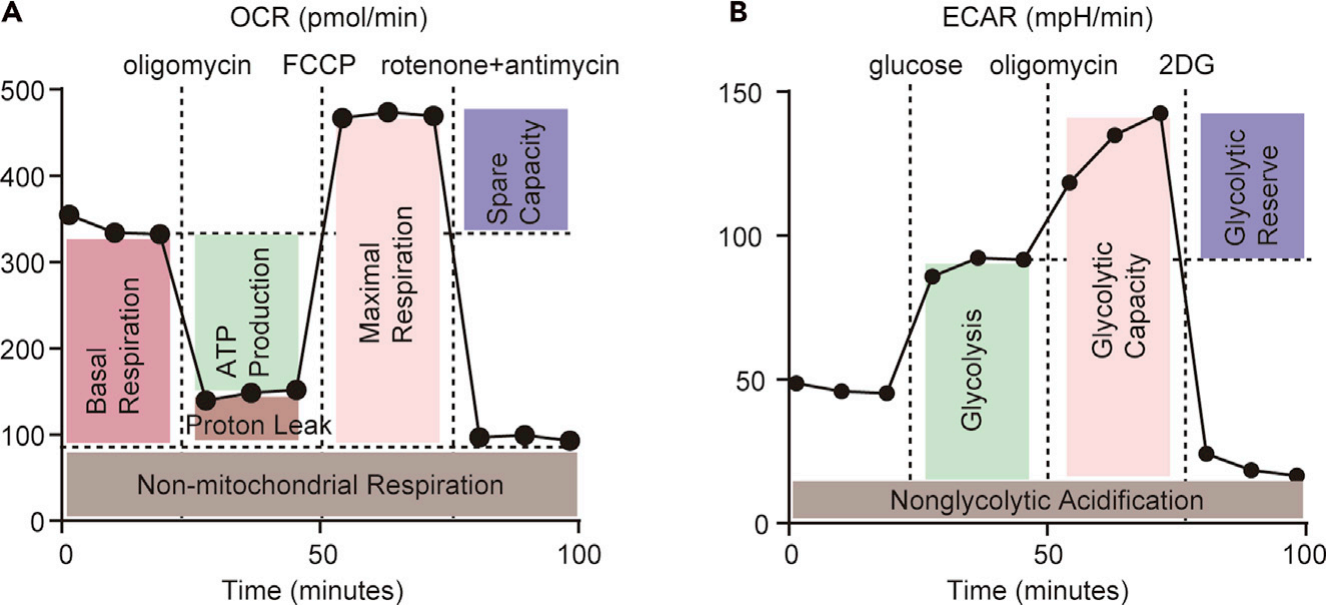
Statistical analysis of mitochondrial stress test and glycolytic stress test A schematic of a mitochondrial stress test (A) and glycolytic stress test (B) using the extracellular flux analyzer.

***Note:*** Agilent Seahorse Wave Desktop software includes several programs that can automatically measure Mito Fuel Flex Test, Cell Mito Stress Test, Cell Energy Phenotype Test, Glycolytic Rate Assay, and Real-Time ATP Rate Assay (Figure 7C).

## Limitations

In these protocols, we have described several analytic methods using mouse BMDCs. Because we have analyzed similar assays on various cells, we consider these protocols to be beneficial. Conversely, there are several new reagents and protocols. Therefore, when you perform analyses with a Seahorse extracellular flux analyzer, we recommend checking for new protocols at https://www.agilent.com/en/product/cell-analysis/real-time-cell-metabolic-analysis.

## Troubleshooting

### Problem 1

**Figure 9:**
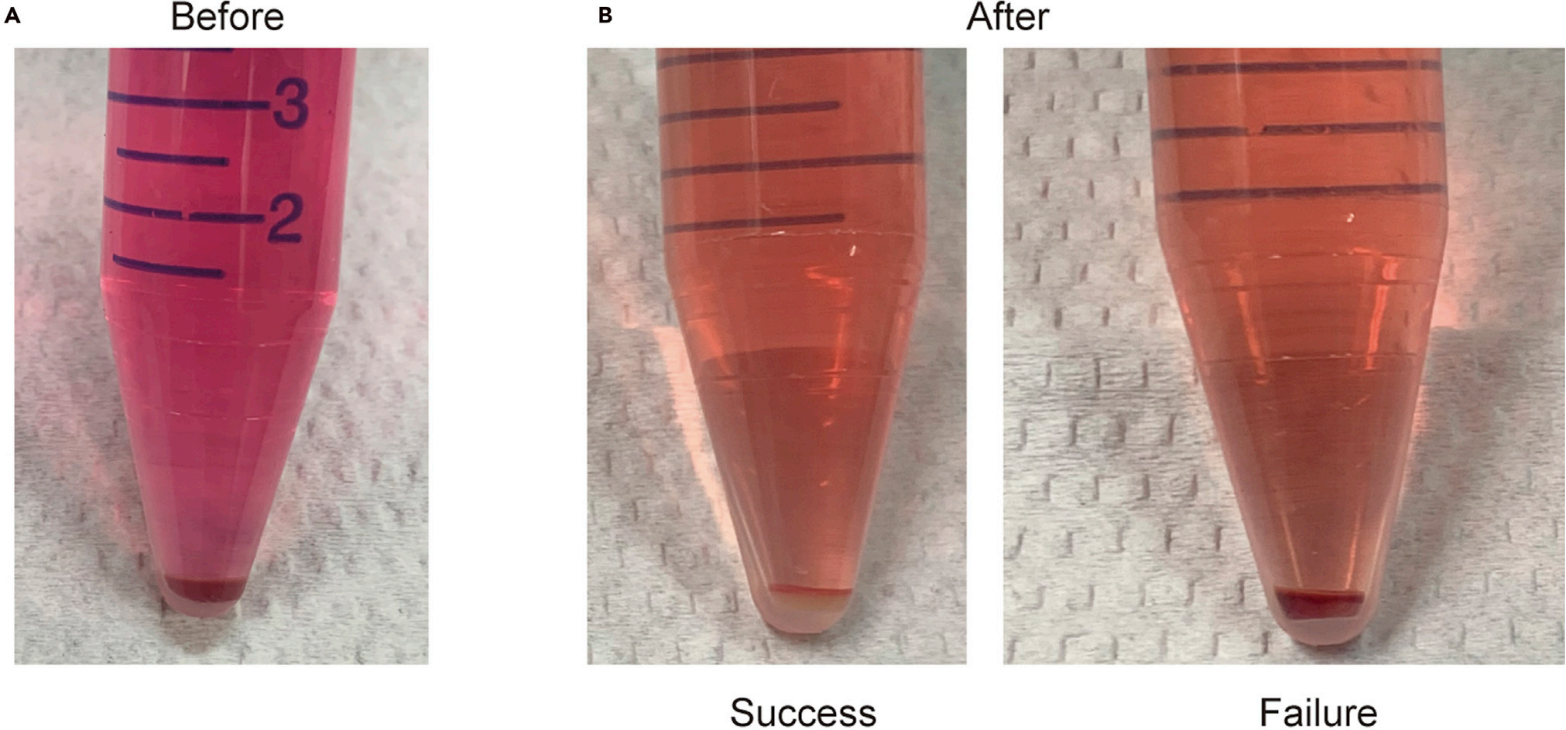
Photograph of bone marrow pellets Bone marrow pellet before RBC lysis (A), after RBC lysis (B).

RBCs are not hemolysed by RBC lysis buffer. Refer to Figure 9 (step 10).

### Potential solution

Extend the mixing time to 60–120 s.
Add 2 mL / body of RBC lysis buffer to the cells.
Remake RBC lysis buffer.

### Problem 2

Bone marrow cells are not properly differentiated into BMDCs (step 15).

### Potential solution

Reduce the number of bone marrow cells seeded onto the culture plate.
Extend the culturing period of the BMDCs.
Increase the concentration of GM-CSF.
Use a different lot of fetal bovine serum.

### Problem 3

The measured value of OCR or ECAR is small or lower than the sensitivity (step 45).

### Potential solution

Check the viability of BMDCs.
Increase the number of BMDCs seeded into an XF24 cell culture microplate.
Improve the condition of the BMDCs.
Prepare the assay medium again.

### Problem 4

ECAR does not increase after the administration of TLR agonist (step 45).

### Potential solution

Increase or decrease the concentration of the TLR agonist.
ECAR may not increase in DCs whose energy metabolism has shifted from mitochondrial respiration to glycolysis by the inhibitor or genetic modification.
Reduce the FCS concentration to 1%–5% because FCS has some buffering capacity.

### Problem 5

The value of OCR and ECAR is not stable (step 45).

### Potential solution

**CRITICAL:** We recommend checking raw data for pO_2_ and pH.

Check the levels of pO_2_ and pH.
When a large number of cells are seeded in a well, an artifact of OCR may be generated due to the hypoxia. If pO2 or pH decreases over time, we recommend to reduce the number of cells or increase the rest time of assay (Gerencser et al., 2009).

## Resource availability

### Lead contact

Further information and requests for resources and reagents should be directed to and will be fulfilled by the lead contact, Kazuhito Gotoh (gotou.kazuhito.712@m.kyushu-u.ac.jp).

### Materials availability

All reagents generated in this study are available from the lead contact with a completed Materials Transfer Agreement.

### Data and code availability

The data that support the findings of this study are available from the lead contact upon reasonable requests.
